# LPS-induced NFκB enhanceosome requires TonEBP/NFAT5 without DNA binding

**DOI:** 10.1038/srep24921

**Published:** 2016-04-27

**Authors:** Hwan Hee Lee, Satoru Sanada, Seung Min An, Byeong Jin Ye, Jun Ho Lee, Young-Kyo Seo, Changwook Lee, Whaseon Lee-Kwon, Christoph Küper, Wolfgang Neuhofer, Soo Youn Choi, Hyug Moo Kwon

**Affiliations:** 1 School of Life Sciences, Ulsan National Institute of Science and Technology, Korea; 2 Division of Nephrology, Japan Community Care Organization Sendai Hospital, Japan; 3 Institute of Physiology, University of Munich, Germany; 4 Department of Medicine, Heidelberg University, Germany

## Abstract

NFκB is a central mediator of inflammation. Present inhibitors of NFκB are mostly based on inhibition of essential machinery such as proteasome and protein kinases, or activation of nuclear receptors; as such, they are of limited therapeutic use due to severe toxicity. Here we report an LPS-induced NFκB enhanceosome in which TonEBP is required for the recruitment of p300. Increased expression of TonEBP enhances the NFκB activity and reduced TonEBP expression lowers it. Recombinant TonEBP molecules incapable of recruiting p300 do not stimulate NFκB. Myeloid-specific deletion of TonEBP results in milder inflammation and sepsis. We discover that a natural small molecule cerulenin specifically disrupts the enhanceosome without affecting the activation of NFκB itself. Cerulenin suppresses the pro-inflammatory activation of macrophages and sepsis without detectable toxicity. Thus, the NFκB enhanceosome offers a promising target for useful anti-inflammatory agents.

Nuclear factor κB (NFκB) transcription factors regulate the expression of several hundred cellular genes involved in a variety of cellular and physiological processes, such as immune and inflammatory responses, developmental processes, cellular growth, and apoptosis^1^,^2^. In mammals, five genes encode proteins for NFκB: RelA (p65), RelB, c-Rel, NFκB1 (p105 which is processed into p50), and NFκB2 (p100 which is processed into p52). The protein products form a variety of homo- and hetero-dimers to bind DNA, of which the p65:p50 heterodimer is most commonly found. The key regulatory event in the activation of the p65:p50 heterodimeric NFκB is the phosphorylation of IκB proteins by the IκB kinase complex, which leads to IκB protein ubiquitination and subsequent degradation^2^,^3^. Removal of IκB leads to the release of cytoplasmic p65:p50 heterodimer, which then moves into the nucleus, associates with transcriptional cofactors p300 and CREB binding protein (CBP), and drives the expression of target genes. Recent studies have shown that DNA bound NFκB initiates the formation of distinct enhanceosomes in a target gene-specific manner^4^,^5^,^6^,^7^. NFκB is persistently active in a number of human diseases, including sepsis, cancer, arthritis, chronic inflammation, asthma, neurodegenerative diseases, metabolic disease, and heart disease^8^,^9^,^10^, and thus, effective and safe inhibitors of NFκB would have wide-ranging therapeutic use.

Tonicity-responsive enhancer binding protein (TonEBP), also known as nuclear factor of activated T cells 5 (NFAT5), belongs to the Rel family of transcriptional factors which include NFκB and NFAT^11^,^12^. TonEBP was initially identified as the central regulator of cellular response to hypertonic stress^11^,^13^,^14^,^15^. Recent studies have demonstrated that TonEBP is involved in the M1 activation of macrophages by promoting the expression of pro-inflammatory genes in response to Toll-like receptor activation^16^,^17^. TonEBP haplo-deficiency is associated with dramatically reduced inflammation and pathology in mouse models of rheumatoid arthritis^18^ and atherosclerosis^19^.

In order to understand molecular basis of the TonEBP function in inflammation, we investigated TonEBP action in the induction of pro-inflammatory genes in response to LPS. Surprisingly, we discover that DNA binding of TonEBP is dispensable. Instead, TonEBP is required for the activation of NFκB by way of recruiting transcriptional cofactor p300 through protein-protein interactions. Our data demonstrate that TonEBP is an essential component of the LPS-induced NFκB enhanceosome critical for expression of pro-inflammatory genes.

## Results

### TonEBP promotes macrophage activation and sepsis

**Figure 1:**
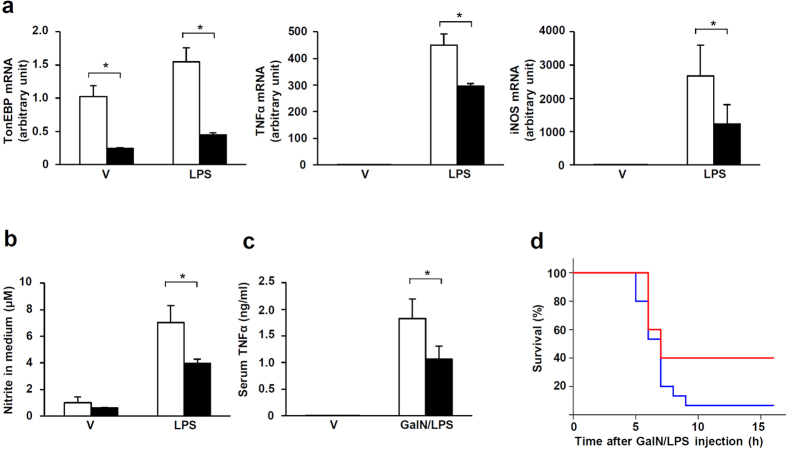
Reduced inflammation and septic death in mice with myeloid-specific deletion of the TonEBP gene. (**a**) Peritoneal macrophages (PECs) isolated from *TonEBP*^*flx/flx*^, *LysM-cre* (solid bars) and their *TonEBP*^*flx/flx*^ littermates (open bars) were stimulated for 6 h with LPS or vehicle (V). mRNA expression for TonEBP, TNFα and iNOS was measured by RT Q-PCR. (**b**) PECs were treated for 24 h with LPS or vehicle (V). Nitrite was measured using Griess reagent from the media. Mean + SD, n = 5. *p < 0.05. (**c**) *TonEBP*^*flx/flx*^, *LysM-cre* mice (solid bars) and their *TonEBP*^*flx/flx*^ littermates (open bars) were intraperitoneally injected with D-galactosamine (700 mg/kg) plus LPS (150 μg/kg) (GalN/LPS) or vehicle (V). After 1 h, TNFα was measured using ELISA from serum samples. Mean + SD, n = 4. *p < 0.05. (**d**) *TonEBP*^*flx/flx*^, *LysM-cre* (red line) and their *TonEBP*^*flx/flx*^ littermates (blue line) were intraperitoneally injected with GalN/LPS. The animals were monitored for 16 h for survival n = 15.

Macrophage activation is a hallmark of inflammation, and NFκB is a central regulator of pro-inflammatory macrophage activation^20^. In order to explore the role of TonEBP in macrophage activation, we obtained a line of mice with myeloid-specific deletion of the TonEBP gene by crossing the line in which the exon 4 of the TonEBP gene was flanked by lox P sequences (*TonEBP*^*fl*^ allele)^21^ with the line expressing the cre recombinase in myeloid cells using the promoter of the lysozyme 2 gene (LysM-cre)^22^. Peritoneal macrophages (PECs) prepared from *TonEBP*^*flx/flx*^, *LysM-cre* mice showed a dramatically reduced TonEBP mRNA expression compared to those prepared from their *TonEBP*^*flx/flx*^ littermates (Fig. 1a). TonEBP mRNA expression in other tissues such as liver and brain was normal consistent with myeloid-specific deletion of the TonEBP gene (data not shown). When stimulated with LPS, PECs from the *TonEBP*^*flx/flx*^, *LysM-cre* mice showed significantly reduced expression of NFκB-dependent pro-inflammatory genes TNFα and iNOS (Fig. 1a), and NO production (Fig. 1b) in response to LPS. In response to D-galactosamine and LPS administration, which was used to produce a mouse model of sepsis^23^, the rise in serum TNFα levels were reduced by ~40% in the *TonEBP*^*flx/flx*^, *LysM-cre* mice compared to their *TonEBP*^*flx/flx*^ littermates (Fig. 1c). Furthermore, severity of sepsis measured by ensuing death was reduced in the *TonEBP*^*flx/flx*^, *LysM-cre* animals (Fig. 1d). These data show that myeloid-specific deletion of the TonEBP gene results in blunted macrophage activation and septic shock in association with inflammatory responses.

### TonEBP binds to the TNFα promoter without direct interaction with DNA

**Figure 2:**
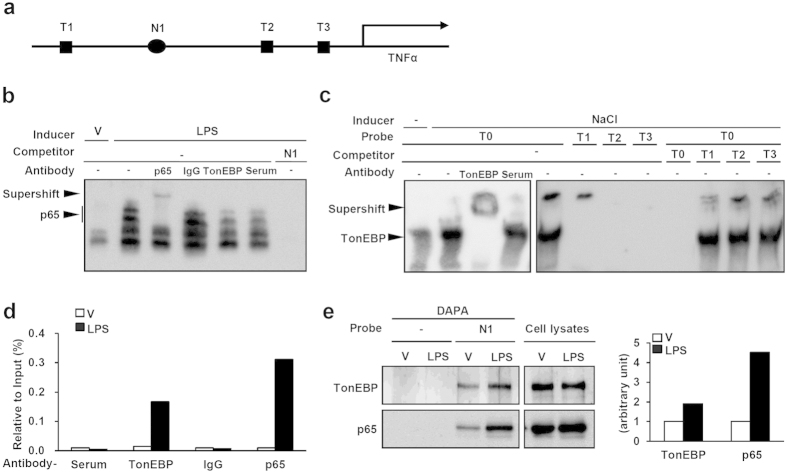
TonEBP binds to the κB site of the TNFα promoter without direct interaction with DNA. (**a**) Schematic representation of putative binding sites of TonEBP (T1, T2, T3) and NFκB (N1) in the TNFα promoter region. DNA sequence of the sites is provided in Methods. (**b**) Nuclear extracts were prepared from RAW264.7 cells stimulated for 1 h with LPS (100 ng/ml) or vehicle (V). EMSA was performed using the nuclear extracts and biotin-labeled N1 probe. Where indicated, anti-p65 IgG (p65) or normal IgG (IgG) was added to supershift p65-DNA complex; and-TonEBP serum (TonEBP) or normal rabbit serum (Serum) to supershift TonEBP-DNA complex. In the last lane, 50 times concentration of unlabeled N1 was added for competition. (**c**) Nuclear extracts were prepared from RAW264.7 cells cultured for 24 h in hypertonic medium containing extra 75 mM NaCl (NaCl) or control, isotonic medium (−). EMSA was performed using the nuclear extracts and biotin-labeled probes: T0 (positive control for TonEBP binding), T1, T2 and T3. Anti-TonEBP antibody and normal rabbit serum were used to supershift TonEBP-DNA complex. In the last 4 lanes, 50 times concentration of unlabeled probe was added for competition. (**d**) RAW264.7 cells were treated for 1 h with LPS or vehicle (V). ChIP was performed using normal rabbit serum, anti-TonEBP serum, normal IgG, and anti-p65 IgG. The precipitates were quantified for the N1 region using Q-PCR. A representative set of four independent sets of experiments is shown. (**e**) Cell extracts were prepared from RAW264.7 cells treated for 1 h with LPS or vehicle (V). Biotin-labeled N1 probe was used to perform DAPA. DAPA samples and cell lysates were immunoblotted for TonEBP and p65. Images of immunoblots are shown on the left, and intensity ratios of TonEBP and p65 in DAPA/cell lysate are plotted on the right. A representative set of four independent sets of experiments is shown.

In macrophages TonEBP is induced by Toll-like receptor engagement and activates many genes by direct binding to their promoters including that of TNFα^16^. Figure 2a shows a schematic representation of 1.6 kb upstream of the mouse TNFα gene where three putative binding sites for TonEBP (T1, T2, and T3) and a putative NFκB binding site (N1) are located. In order to understand molecular action of TonEBP, we first examined the affinity of TonEBP and NFκB to the sites using electrophoretic mobility shift assay (EMSA) of nuclear extracts prepared from RAW264.7 macrophage cell line. The N1 probe detected several prominent bands which showed up after LPS stimulation (Fig. 2b). All of these bands were competed away by excess cold probe, but only the top two bands were supershifted by p65 antibody but not by TonEBP antiserum indicating that they represented p65-containing NFκB molecules. These data demonstrate that N1 is a functional κB sequence and p65-containing NFκB molecules have specific affinity to N1.

The macrophage nuclear extracts displayed specific binding to a tonicity-responsive enhancer (TonE) sequence (T0) which was enhanced by hypertonicity, supershifted by TonEBP antiserum, and competed away by excess cold probe demonstrating that the band represented DNA-bound TonEBP molecules (Fig. 2c). On the other hand, none of the putative TonEBP binding sequences T1, T2, and T3 displayed binding to the band, and were unable to compete with T0 for TonEBP binding. Thus, T1, T2, and T3 are not a functional TonE and TonEBP does not have high affinity for the DNA sequence in the promoter region.

We next performed chromatin immunprecipitation (ChIP) to investigate NFκB and TonEBP interaction to the promoter *in situ*. p65 bound to the N1 region in LPS-dependent manner (Fig. 2d) as expected from the EMSA data above. Surprisingly, TonEBP also displayed LPS-dependent binding to the region. Since TonEBP did not have affinity to the sequence in the region, we asked whether there was a protein-protein interaction between TonEBP and p65. To address this question, we performed DNA affinity purification analysis (DAPA) of the cell lysates using a biotin-labeled N1 probe. Both NFκB and TonEBP displayed LPS-dependent binding to the N1 probe (Fig. 2e) suggesting that there is a protein-protein interaction between them.

### TonEBP interacts with p65 through Rel-homology domains (RHDs)

**Figure 3:**
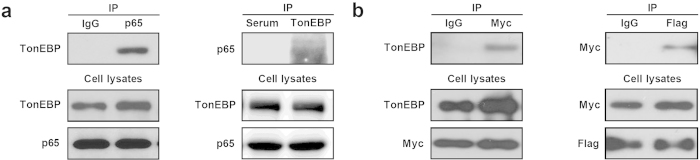
TonEBP interacts with p65. (**a**) MEF cell lysates were immunoprecipitated (IP) with normal IgG or anti-p65 IgG (left), or normal serum or anti-TonEBP serum (right). The immunoprecipitates and cell lysates were immunoblotted for TonEBP and p65 as indicated. (**b**) COS-7 cells were co-transfected with Myc-p65 and Flag-TonEBP. Cell lysates were immunoprecipitated with normal IgG or anti-Myc IgG (left), or anti-Flag IgG (right). The immunoprecipitates and cell lysates were immunoblotted for TonEBP, Myc, or Flag as indicated.

**Figure 4:**
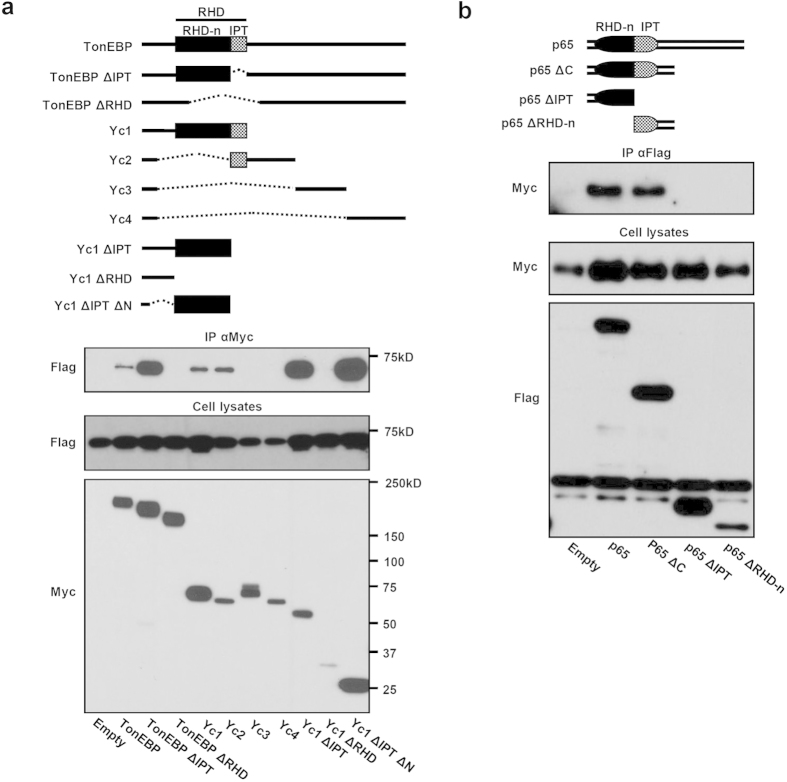
TonEBP interacts with p65 via respective RHDs. (**a**) Schematic representation of human TonEBP and its recombinant fragments. RHD, Rel-homology domain – amino acids 280–544^24^; RHD-n, N-terminal subdomain of RHD involved in DNA contact – amino acids 280–444; IPT, Ig-like, plexins, transcription factors domain with an immunoglobin-like fold involved in dimer formation – amino acids 445–544. COS-7 cells were cotransfected for 24 h with Flag-p65 plus Myc-tagged TonEBP or one of the recombinant TonEBP fragments shown. Cell lysates were immunoprecipitated (IP) with anti-Myc antibody. (**b**) Schematic representation of p65 and its fragments. COS-7 cells were cotransfected for 24 h with Myc-Yc1 plus Flag-tagged p65 or one of its fragments shown. Cell lysates were immunoprecipitated with anti-Flag antibody.

Molecular basis of the TonEBP-p65 interaction was investigated. Co-immunoprecipitation experiments revealed that both endogenous proteins (Fig. 3a) and over-expressed proteins (Fig. 3b) could be mutually pulled down by each other. In order to map sites of interaction, we produced various recombinant proteins of TonEBP and p65 (Fig. 4). Analyses of the recombinant proteins suggest that the RHDs are critical for both. TonEBP constructs without RHD – TonEBP ΔRHD and Yc1 ΔRHD – did not show interaction with p65, while those of intact RHD such as Yc1 and partial RHD such as TonEBP ΔIPT did (Fig. 4a). In addition, protein product of a mutant TonEBP allele lacking the N-terminal half of RHD did not bind p65 (see below). Likewise, p65 constructs with its RHD domain partially deleted (p65 ΔIPT and p65 ΔRHD-n) did not interact with TonEBP (Fig. 4b).

### TonEBP stimulates NFκB activity independent of DNA binding

**Figure 5:**
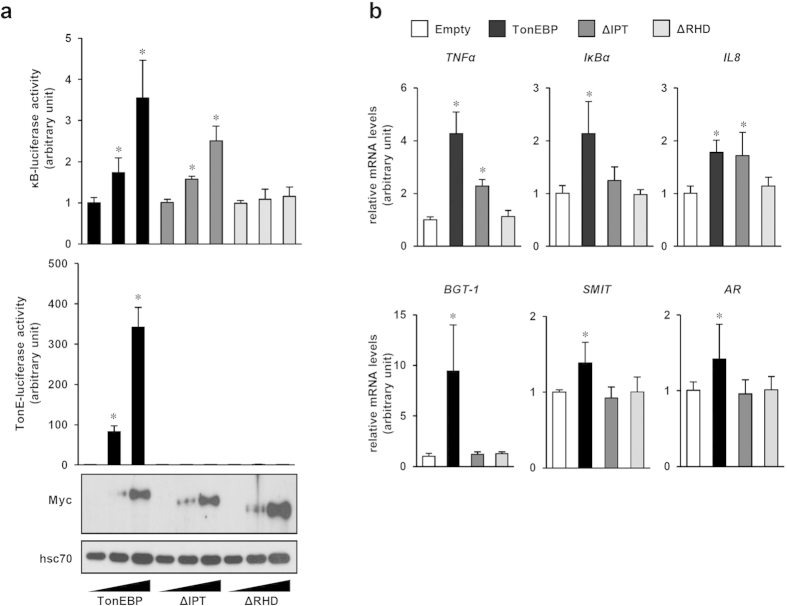
Stimulation of NFκB by TonEBP requires the TonEBP-p65 interaction. (**a**) COS-7 cells were transfected with increasing amount of Myc-tagged TonEBP, TonEBPΔIPT (ΔIPT) or TonEBPΔRHD (ΔRHD) along with κB-luciferase or Ton E-luciferase reporter construct. Cells were stimulated for 6 hours with LPS (100 ng/ml) before measuring luciferase activity. The activity of luciferase is shown relative to those cells transfected with empty expression vector. Expression of TonEBP, TonEBPΔIPT and TonEBPΔRHD protein were examined by immunoblotting with anti-Myc antibody. (**b**) HEK293 cells were transfected with the constructs indicated at the top. mRNA expression was examined by RT Q-PCR for NFκB-target genes TNFα, IκBα, and IL8, and TonEBP-target genes BGT-1, SMIT, and AR. Data are normalized to those transfected with empty vector. Mean + SD, n = 5 or 6. *p < 0.05.

In HEK293 cells, over-expression of TonEBP resulted in a stimulation of both κB-driven and TonE-driven luciferase (Fig. 5a), and expression of NFκB- and TonEBP-target genes (Fig. 5b) as expected. On the other hand, TonEBP ΔRHD, which did not interact with p65 (Fig. 4a) and could not bind DNA^24^, did not stimulate neither κB-driven nor TonE-driven gene expression (Fig. 5). TonEBP ΔIPT lacks the dimerization domain and does not bind DNA^24^. Interestingly, TonEBP ΔIPT retained its ability to interact with p65 (Fig. 4a) and stimulated κB-driven gene expression but not TonE-driven gene expression (Fig. 5). These observations demonstrate that TonEBP stimulates NFκB independent of DNA binding.

**Figure 6:**
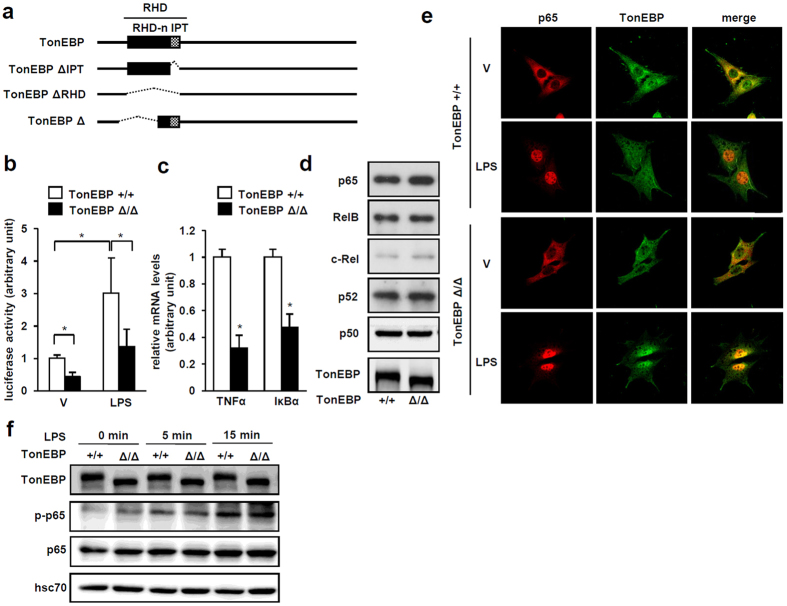
Reduced NFκB activity in TonEBP^Δ/Δ^ MEF cells despite normal expression and regulation of NFκB. (**a**) Schematic representations of TonEBP, TonEBP ΔIPT, TonEBP ΔRHD (see Fig. 3), and protein product of the TonEBP Δ allele which lacks exon 6 and 7 leading to an in-frame deletion of a portion of RHD-n as indicated. (**b**) TonEBP^+/+^ or TonEBP^Δ/Δ^ MEF cells were transfected with the κB-luciferase construct. Cells were treated with LPS (100 ng/ml) or vehicle (V) for 6 hours before measuring luciferase activity. (**c**) Cells were treated with LPS for 6 hours. TNFα and IκBα mRNA were quantified by RT Q-PCR. Mean + SD, n = 4. *p < 0.05. (**d**) Cells were immunoblotted for p65, RelB, c-Rel, p52, p50 and TonEBP. (**e**) Cells grown on coverslips were treated with LPS or vehicle (V), and immunostained for p65 (red) and TonEBP (green). Co-localization is shown in orange in merged images. (**f**) Cells were treated with LPS for 0, 5, and 15 min as indicated, and immunoblotted for TonEBP, serine 276 phosphorylated p65 (p-p65), p65, and hsc70.

**Figure 7:**
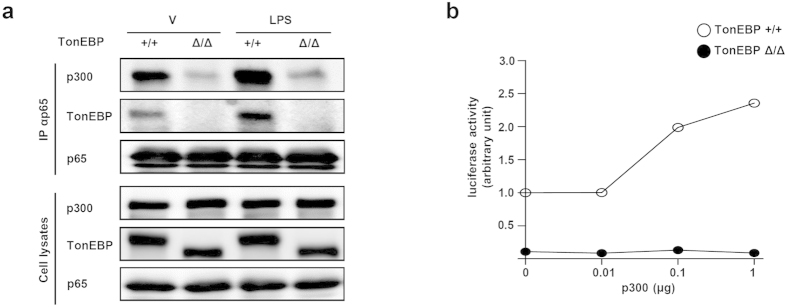
The TonEBP Δ protein is unable to recruit p300 co-activator to p65. (**a**) TonEBP^+/+^ or TonEBP^Δ/Δ^ MEF cells were treated with LPS or vehicle (V). Lysates of TonEBP^+/+^ or TonEBP^Δ/Δ^ MEF cells were immunoprecipitated (IP) using anti-p65 antibody followed by immunoblotting for p300, TonEBP and p65. (**b**) Cells were transfected with increasing amount of a p300 expression plasmid along with the κB-luciferase reporter. After 6 h treatment with LPS, luciferase was measured. A representative of three independent experiments is shown.

The mouse TonEBP Δ allele was created by deletion of exon 6 and 7^15^. The deletion results in an in-frame deletion of 128 amino acids in the N-terminal portion of RDH as depicted in Fig. 6a. Interestingly, mouse embryonic fibroblast (MEF) cells established from *TonEBP*^Δ*/*Δ^ mice showed reduced κB-driven luciferase expression both in basal conditions and after LPS stimulation (Fig. 6b). Expression of NFκB-target genes TNFα and IκBα in response to LPS was also reduced in these cells (Fig. 6c) consistent with reduced NFκB activity. We investigated molecular basis for the reduced NFκB activity. The *TonEBP*^Δ*/*Δ^ MEF cells expressed the TonEBP Δ protein which migrated faster than the wild type TonEBP protein (Fig. 6d). In these cells, expression of the NFκB subunits – p65, RelB, c-Rel, p52, and p50 – was normal. Nuclear localization of p65 and TonEBP in response to LPS looked normal (Fig. 6e). In addition, serine 276 phosphorylation of p65 in response to LPS was normal in the *TonEBP*^Δ*/*Δ^ MEF cells (Fig. 6f). Finally, we asked whether reduced TonEBP expression affected DNA binding of p65. Knock-down of TonEBP did not affect DNA binding of p65 based on EMSA and DAPA (Supplementary Fig. 1). Thus, NFκB activity was reduced in the *TonEBP*^Δ*/*Δ^ MEF cells despite normal expression of NFκB subunits, nuclear translocation, phosphorylation, and DNA binding of p65 in response to LPS. The reduced NFκB activity can be explained by the inability of the mutant protein to interact with p65 (Fig. 7a) providing further support to the notion that TonEBP stimulates NFκB independent of DNA binding, i.e, *via* protein-protein interaction.

### TonEBP is required for the recruitment co-activator p300 to NFκB: LPS-induced NFκB enhanceosome

We asked how the TonEBP-p65 interaction led to stimulation of the NFκB activity. Two possibilities were explored. One was recruitment of the large and powerful transactivation domain (TAD) of TonEBP^25^. To test this, we generated a fusion protein of p65 and the TAD. The fusion protein displayed a markedly elevated transcriptional activity which was gradually reduced as the TAD domain was serially deleted from the C-terminus (Supplementary Fig. 2a,b) as reported earlier^25^. This observation suggests that the TAD of TonEBP enhance transactivation by p65 bound to DNA. On the other hand, over-expression of Yc1 (see Fig. 4a), which was expected to compete away TAD-containing TonEBP and reduce transactivation of NFκB, did not inhibit NFκB (Supplementary Fig. 2c) suggesting that there should be other pathways of transactivation by TonEBP.

We explored the possibility that TonEBP was involved in the recruitment of transcriptional co-activators such as p300. p300 is an acetyltransferase involved in p65 acetylation which is essential in the assembly of NFκB enhanceosome^26^. When p65 was immunoprecipitated, both TonEBP and p300 were also brought down (Fig. 7a) suggesting that the all the three molecules were in a complex. The interaction increased in response to LPS (Fig. 7a) in correlation with increased nuclear localization of p65 (Fig. 6e). Of note, the TonEBP Δ protein did not interact with p65 and, thereby, the amount of p300 brought down with p65 was dramatically lower in the *TonEBP*^Δ*/*Δ^ MEF cells (Fig. 6a, 2^nd^ and 4^th^ lane). The inability of the TonEBP Δ protein to interact with p65 and recruit p300 explains the reduced NFκB activity in the *TonEBP*^Δ*/*Δ^ MEF cells (Fig. 6). These data indicate that the TonEBP-p65 interaction was critical for the recruitment of p300 to p65. This was further supported by the observation that increased expression of p300 did not lead to increased κB-driven luciferase expression in the *TonEBP*^Δ*/*Δ^ MEF cells (Fig. 7b). Thus, the inability of the TonEBP Δ protein to transactivate NFκB was due to its inability to interact with p65, i.e., inability to form the NFκB enhanceosome.

The TonEBP containing enhanceosome complex was characterized further. Knockdown of p300 led to reduced recruitment of both p300 and TonEBP to p65 (Supplementary Fig. 3a), while knockdown of p65 did not affect the association between p300 and TonEBP (Supplementary Fig. 3b). These observations suggest that preformed TonEBP-p300 heterodimer is recruited to p65 after its nuclear translocation in response to LPS. Knockdown of TonEBP led to reduced p300 recruitment to p65 (Supplementary Fig. 3c), as expected. Of note, ChIP experiments revealed that the LPS-dependent assembly of NFκB enhanceosome on the promoter of the TNFα promoter, as measured by recruitment of p65, Sp1 and Pol II to the N1 site^27^,^28^, was reduced after TonEBP knockdown (Supplementary Fig. 4). Thus, TonEBP deficiency is associated with not only lower recruitment of p300 to p65 but also reduced assembly of the NFκB enhanceosome on the promoter leading to lower NFκB activity observed under these conditions.

### Cerulenin disrupts the p65-TonEBP-p300 interaction and inhibits NFκB without toxicity

**Figure 8:**
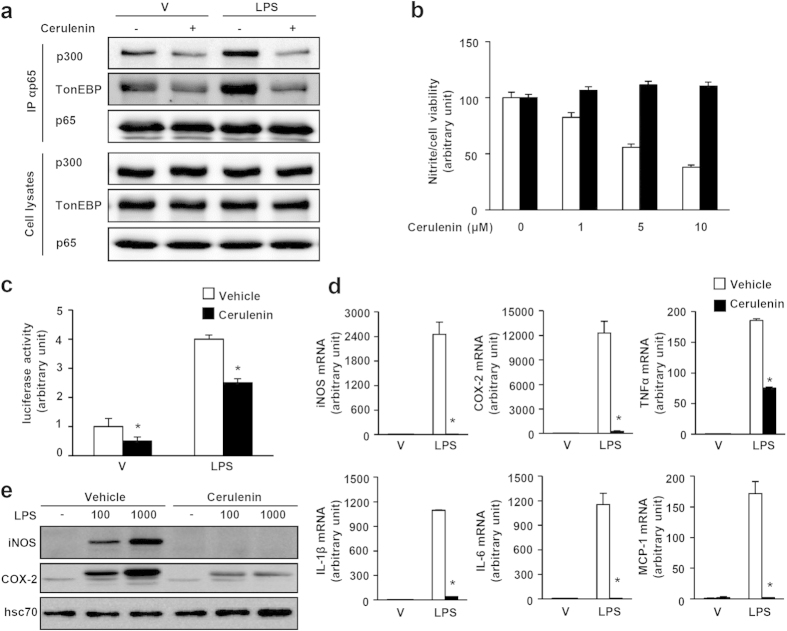
Cerulenin breaks up the TonEBP-p300-p65 interaction and reduces inflammation. (**a**) MEF cells were treated without or with cerulenin (10 μM) for 1 h followed by vehicle (V) or LPS (100 ng/ml) for an additional 1 h. Cell lysates were immunoprecipitated (IP) using anti-p65 antibody. The immunoprecipitates and cell lysates were immunoblotted for p300, TonEBP and p65. (**b**) PECs were treated with 0 to 10 μM cerulenin as indicated for 1 h, followed by LPS for 24 h. Nitrite in the medium (open bars) and cell viability based on reduction of MTT (solid bars) were measured. (**c**) PECs transfected with the κB-luciferase construct were treated with vehicle or cerulenin (10 μM) for 1 h, followed by vehicle (V) or LPS for an additional 6 h. Luciferase activity was measured from cell lysates. Mean + SD, n = 4. *p < 0.05. (**d**) PECs were treated as in (c) and mRNA expression for iNOS, COX-2, TNFα, IL-1β, IL-6 and MCP-1 was measured by RT Q-PCR. Mean + SD, n = 5. *p < 0.05. (**e**) PECs pre-treated for 1 h with vehicle or cerulenin (10 μM) were stimulated for 24 h with 0, 100, and 1,000 ng/ml of LPS. Expression of iNOS and COX-2 was visualized by immunoblotting.

From a screening of natural compounds for inhibition of NFκB, we discovered that cerulenin, an inhibitor of fatty acid synthase^29^, disrupted the p65-TonEBP-p300 interaction. Cerulenin reduced co-precipitation of TonEBP and p300 in p65 immunoprecipitation assays both under basal conditions and after LPS treatment (Fig. 8a).

**Figure 9:**
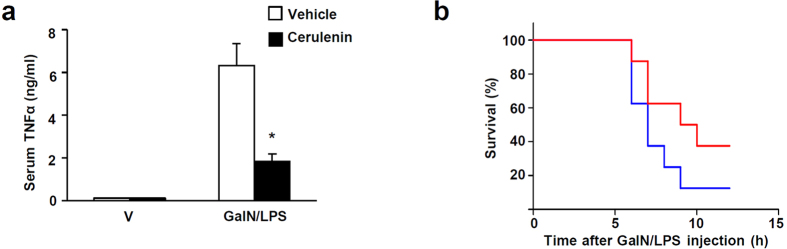
Cerulenin reduces inflammation and septic death in mice. (**a**) Mice were intraperitoneally injected with vehicle or cerlenin (60 mg/kg body weight). After 1 h, vehicle or GalN/LPS was administered as in Fig. 1. After another hour, TNFα was measured from serum samples. Mean + SD, n = 5. *p < 0.05. (**b**) Animals pre-treated with vehicle (blue line) or cerulenin (red line) were administered with GalN/LPS as in (**a**). The animals were monitored for survival n = 8.

We characterized cerulenin action in molecular detail. Cerulenin inhibited NO production in response to LPS in a dose-dependent manner without compromising cell viability (Fig. 8b). The lack of cytotoxicity by cerulenin contrasts with the toxicity of BAY11-7082, a protein kinase inhibitor: while both compounds inhibited NO production with comparable efficacy, cerulenin displayed no detectible toxicity unlike BAY11-7082 which displayed a dose-dependent decrease in viability (Supplementary Fig. 5). The reduced NO production was associated with reduced NFκB activity based on reduced κB-driven luciferase expression (Fig. 8c) and reduced expression of NFκB-target genes (Fig. 8d) and their proteins (Fig. 8e). As expected, cerulenin potently inhibited systemic inflammation and septic death (Fig. 9). Of great interest, the reduced NFκB activity was not associated with changes in the nuclear localization, DNA binding, or phosphorylation of p65 (Supplementary Fig. 6). In addition, cerulenin did not affect the expression of TonE-driven luciferase and TonEBP target genes in response to hypertonicity (data not shown). Taken together, the data provide compelling evidence that cerulenin prevents inflammation by inhibiting NFκB as it specifically disrupts the p65-TonEBP-p300 interaction. As such, cerulenin displayed lower, if any, toxicity.

## Discussion

**Figure 10:**
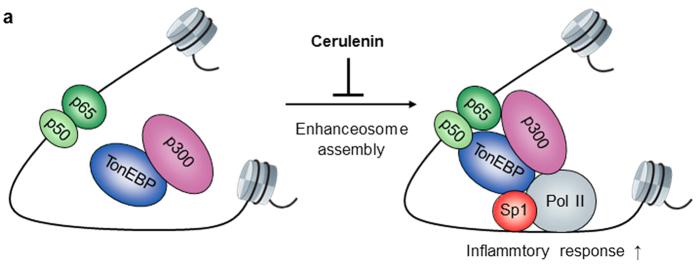
Schematic model of NFκB enhanceosome assembly in response to inflammatory stimulation and its disruption by cerulenin. See text for details.

Although the molecular interaction between p300 and p65 subunit of NFκB was recognized early on^30^,^31^, coordinated recruitment of p300 and p65 to the promoters of TNFα and IL-12 genes of macrophages in response to LPS was demonstrated fairly recently^32^. Our date presented here reveal that TonEBP is required for the molecular interaction between p300 and p65. Preformed nuclear TonEBP-p300 complex unites with p65 after it enters the nucleus in response to LPS (Fig. 10). This p65-TonEBP-p300 complex is unique in that increased TonEBP expression leads to higher NFκB activity in correlation with more recruitment of p300; reversely, decreased TonEBP expression leads to lower NFκB activity. This property provides an explanation for the milder inflammation in TonEBP haplo-deficient mice: the spike in serum TNFα in response to LPS is reduced (Supplementary Fig. 7) as in the mice with myeloid TonEBP deficiency (Fig. 1c). The milder inflammation in TonEBP haplo-deficiency is consistent with reduced inflammatory damages observed in this model: dramatically reduced rheumatoid arthritis^18^ and the size of atherosclerotic lesions reduced to one fifth^19^.

TonEBP was originally identified based on its specific DNA binding to the TonE sequence^11^. *In vivo* footprinting analyses revealed that DNA binding of TonEBP to the TonE sites in the promoter regions of its target genes temporally correlated with transcriptional stimulation in response to hypertonicity^33^,^34^. ChIP experiments have shown that the induction of TNFα in response to LPS is associated with TonEBP recruitment to its promoter (Fig. 2)^16^. The data presented here demonstrate that the recruitment is independent of DNA binding. Rather, TonEBP is recruited through a protein-protein interaction with NFκB. We recently reported analogous DNA binding-independent recruitment of TonEBP to the promoter of the peroxisome proliferator-activated receptor γ gene in association with the suppression of the promoter^35^.

The histone acetyl transferase activity of p300 is critical for the formation of NFκB enhanceosome^36^: After recruitment to p65 bound to DNA, p300 acetylates histones leading to opening/remodeling of chromatin and binding of proximal factors such as Sp1 and RNA polymerase II. The data presented here are consistent with this model in that TonEBP deficiency results in not only reduced recruitment of p300 to the TNFα promoter but also other components of the NFκB enhanceosome such as Sp1 and RNA polymerase II. Thus, TonEBP-dependent recruitment of p300 is a key early step in the formation of NFκB enhanceosome.

NFκB-mediated inflammation is involved in the pathogenesis of a wide range of diseases including cancer^3^, metabolic and vascular disease^9^, and even viral infection^37^. Much of the anti-inflammatory activity of the widely used glucocorticoids is due to blockage of NFκB activity^38^. Genetic and pharmacological inhibition of NFκB reverses insulin resistance in animal models^39^. A recent clinical trial showed that salicylate, which inhibits NFκB, improved glycemia in patients with type 2 diabetes and decreased inflammatory mediators^40^. Effective inhibitors of NFκB with minimal side effects would be quite useful for therapeutic use against the diverse inflammatory diseases.

A natural lipid analog cerulenin, (2R)(3S)-2,3-epoxy-4-oxo-7,10-dodecaldienoylamide, is an irreversible inhibitor of fatty acid synthase. Here we discover that cerulenin is a powerful inhibitor of NFκB-mediated inflammation and sepsis. Cerulenin acts by way of disrupting the p65-TonEBP-p300 complex without affecting the expression, DNA binding, and regulation of p65 itself (Fig. 10). In this regard it is distinct from most of the known NFκB inhibitors which target broad cellular processes such as protein phosphorylation, proteasome, nuclear import machinery, or activation of nuclear receptors. Animals tolerate high doses of cerulenin for many weeks^41^ consistent with little cellular toxicity observed in this study. Cerulenin offers an attractive opportunity to develop new class of effective and safe anti-inflammatory agents.

## Methods

### Animals

All the methods involving live mice were carried out in accordance with the approved guidelines. All experimental protocols were approved by Institutional Animal Care and Use Committee of the Ulsan National Institute of Science and Technology (UNISTACUC-12-15-A).

Mice carrying the *loxP*-targeted TonEBP gene (*TonEBP*^*flx/flx*^) were reported previously^21^. Transgenic mice expressing Cre recombinase under the control of either the myeloid-specific lysozyme M (LysM) promoter were purchased from The Jackson Laboratory (Bar Harbor, ME, USA). *TonEBP*^*flx/flx*^ and *LysM-cre* mice crossed to yield mice with specific targeted deletion of TonEBP in macrophages (*TonEBP*^*flx/flx*^, *LysM-cre*). For septic shock, *TonEBP*^*flx/flx*^, *LysM-cre* mice and their *TonEBP*^*flx/flx*^ littermates were intraperitoneally injected with D-galactosamine (700 mg/kg; Sigma Aldrich) plus LPS (150 μg/kg; Sigma Aldrich). After injection, animals were monitored for 16 h for survival.

### Cells and Reagents

Macrophage cell line RAW264.7 cells, mouse embryo fibroblasts (MEFs), COS-7 cells and HEK293 cells were cultured in Dulbecco’s Modified Eagle’s Medium (DMEM) containing 10% fetal bovine serum (FBS; Thermo fisher scientific Inc, Waltham, MA, USA) and penicillin/streptomycin (100 U/ml and 100 μg/ml; GE healthcare life sciences, Logan, UT, USA). RAW264.7 cells were collected by scraping with a rubber policeman, while MEFs, COS-7 cells and HEK293 cells were collected using trypsin/EDTA (Invitrogen, Carlsbad, CA, USA). Peritoneal macrophages derived from *TonEBP*^*flx/flx*^ and *TonEBP*^*flx/flx*^*, LysM-cre* mice were cultured in RPMI containing 10% fetal bovine serum, penicillin/streptomycin (100 U/ml and 100 μg/ml). Cells were maintained at 37 °C in incubator with 5% CO_2_. Cells were pretreated with cerulenin and BAY 11-7082 (Sigma Aldrich, USA) for 1 h and exposed to lipopolysacharide (LPS; Sigma Aldrich). Anti-p65, RelB, c-Rel and p52 antibodies from NFκB family sample kit (4776, Cell Signalling Technologies, Berkeley, CA, USA), anti-p50 (sc8414, SantaCruz Biotechnology, Santa Cruz, CA, USA City), ser 276 phosphorylated p65 (sc101749, SantaCruz Biotechnology), p300 (sc584, SantaCruz Biotechnology) and LaminB (sc6217, SantaCruz Biotechnology) antibodies, anti-Hsc70 (200-301-A28, Rockland, Gilbertsville, PA, USA) and anti-TonEBP antibody^10^ were used for immunoblotting. Cells were transfected with lipofectamine 2000 (Invitrogen, Carlsbad, CA, USA). siRNA duplexes were purchased from Integrated DNA Technologies (Coralville, IA, USA).

### Immunoblot assay

Cell lysis for protein extraction was performed as previously described^42^. Protein concentration was measured by BCA protein assay system (Pierce, Rockford, IL, USA). Equal amounts of protein from each sample were separated by SDS-PAGE and immunoblotted using specific primary antibodies. HRP-conjugated mouse, rabbit and goat secondary antibodies were used for detection. The antigen-antibody binding was detected by enhanced chemiluminescence Western blotting detection reagents (GE healthcare life sciences).

### RNA isolation and reverse transcription quantitative PCR (RT Q-PCR)

Total RNA was isolated using the TRIzol reagent (Invitrogen) according to the manufacturer’s instructions. cDNA was synthesized by M-MLV reverse transcriptase (Promega, Madison, WI, USA). After reverse transcription, Q-PCR was performed using SYBR Green I Master and LightCycler 480 II (Roche, Rotkreuz, Switzerland). Measured cycle threshold (Ct) values were normalized with cyclophilin A and they were expressed as fold-over control samples.

### Immunohistocytochemistry

The cells were grown on glass coverslips and fixed with 4% paraformaldehyde in PBS (pH 7.4) for 20 min at 4 °C. Cells were permeabilized with 0.3% Triton-X 100 in PBS for 30 min and blocked with PBS containing 3% goat serum and 1% bovine serum albumin for 1 h at room temperature. After incubation with rabbit anti-TonEBP and rabbit anti-p65 overnight at 4 °C, the cells were washed with PBS and treated with goat anti-mouse or anti-rabbit Alexa Fluor 488-conjugated and Alexa Fluor 594-conjugated secondary antibodies for 1 h. Cells were washed with PBS and incubated in 0.1 μg/ml Hochest (DAPI) for 30 min. After wash with PBS, coverslips were mounted onto microscope slides. Images were recorded using an Olympus FV1000 confocal fluorescence microscope.

### Nuclear and Cytoplasmic fractionation

Cells were harvested by using scrapper and centrifugation at 500 g. The cell pellet was washed by suspension with PBS. The cell nucleus and cytoplasm were separated by using Nuclear and Cytoplasmic extraction kit (Pierce) according to manufacturer’s instruction. Nuclear fraction was confirmed by Lamin B.

### Electrophoretic mobility shift assay (EMSA)

A commercial kit was used: Lightshift Chemiluminescent EMSA kit (Pierce). 5 μg of nuclear extracts were incubated with poly(dI:dC), binding buffer and 5′ biotinylated DNA (N1 – AAACAGGGGGCTTTCCCTCCTC, T1 – GCTCCGTGGAAAACTCACTTGG, T2 – TGTCCCCAACTTTCCAAACCCT, T3 – ACCAAGGAAGTTTTCCGAGGGTT, T0-TCATAATGGAAAATTCCATGCCA) at room temperature for 20 min. Samples were separated by electrophoresis for 4 h in 4% (40% 29:1 acrylamide/bis solution) gel for TonEBP and 8% gel for p65. The detection was performed according to manufacturer’s instructions.

### Chromatin immunoprecipitation assay

Cells were grown in 10 cm diameter culture dishes and with LPS when indicated. Fixation was performed with 1% formaldehyde at room temperature for 10 min. The fixation was stopped with 0.125 M glycine for 5 min at room temperature. After three washes with cold PBS, cells were collected and lysed in 1 ml of SDS lysis buffer (1% SDS, 10 mM EDTA and 50 mM Tris-HCl pH 8.1) for 10 min on ice. Cell lysates were sonicated (Bioruptor KRB-01, BMS, Tokyo, Japan) for six cycles of 20 s on plus 30 s off with constant frequency and maximum intensity to obtain DNA fragments between 400 and 1,000 bp. Each sample was diluted 10× in dilution buffer (0.01% SDS, 1.1% Triton X-100, 1.2 mM EDTA, 16.7 mM Tris-HCl pH 8.1 and 167 mM NaCl) for immunoprecipitation. Samples were pre-cleared with protein A Sepharose beads (Millipore, Bedford, MA, USA) that were previously pre-adsorbed with salmon sperm DNA for 1 h at 4 °C. Specific antibodies were added after removing the pre-clearing beads: anti-p65 IgG, and normal rabbit IgG (Abcam, Cambridge, UK), anit-TonEBP serum, and normal rabbit serum (Merck millipore, Darmstadt, Germany). After adding antibodies, the lysates were incubated overnight at 4 °C. Protein A Sepharose beads were then added, incubated for 2 h at 4 °C, and then washed with low salt washing buffer (0.1% SDS, 1% Triton X-100, 20 mM Tris-HCl pH 8.1, 2 mM EDTA, and 10 mM NaCl), high salt washing buffer (0.1% SDS, 1% Triton X-100, 20 mM Tris-HCl pH 8.1, 2 mM EDTA and 500 mM NaCl), LiCl washing buffer (0.25 M LiCl, 1% NP-40, 1% deoxycholic acid, 1 mM EDTA and 10 mM Tris-HCl pH 8.1) and twice with final washing buffer (10 mM Tris-HCl pH 8.0 and 1 mM EDTA). To elute the DNA, beads were incubated with elution buffer (1% SDS and 100 mM NaHCO3) for 20 min at 65 °C. To reverse the cross-linking, samples were incubated overnight at 65 °C 200 mM NaCl, 30 min at 37 °C with 50 μg/ml RNase (Pierce) and 2 hr at 45 °C with 100 μg/ml proteinase K. DNA was purified using the QIAGEN PCR purification system. DNA was then subjected to RT-qPCR using primers; 5′-CCCAACTCTCAAGCTGCTCT-3′ and 5′-CTTCTGAAAGCTGGGTGCAT-3′ for TNFα promoter. Immunoprecipitated DNA from each sample was normalized to its respective chromatin input.

### DNA affinity purification assay (DAPA)

Cells were lysed in lysis buffer (20 mM Tris-HCl pH 7.5, 150 mM NaCl and 0.5% Triton-100). 1 mg of extracts were diluted with binding buffer (4 mM Tris-HCl pH 7.5, 20 mM HEPES pH 7.5, 5% glycerol, 170 mM NaCl, 0.5 mM EDTA, 1 mM MgCl_2_ and 0.1% Triton X-100) and incubated overnight with 5’ end biotinylated DNA probe (30 nM) containing a κB site or putative TonE site of mouse TNF-α promoter. They were mixed for 2 hr with 50 μl of streptavidin-coated agarose beads, and protease inhibitors (Roche). Beads were pelleted, washed two times with TE buffer with 100 mM NaCl, two times with binding buffer, once with PBS and then resuspended in 50 μl of Laemmli sample buffer. Precipitated proteins were separated by 7% SDS-PAGE and immunoblotted for TonEBP and p65.

### Luciferase assay

Cells were transfected with either a TonE-driven Photinus luciferase plasmid or a κB-driven luciferase plasmid in pGL4.74 (hRluc/TK, Promega). The Renilla luciferase reporter plasmid (pRL-TK, Promega) was used as a control for transfection efficiency. Luciferase activity after 8 h of stimulation was measured using the Dual-Luciferase Assay System (Promega) according to the manufacturer’s instructions. Luciferase activity was normalized by activity of renilla luciferase.

### Cytokine production

TNFα in supernatants from LPS-stimulated cells or serum samples from D-Galactosamine (GalN)/LPS-injected mice were analyzed by ELISA using a commercial kit (R&D Systems, Minneapolis, MN, USA).

## Additional Information

**How to cite this article**: Lee, H. H. *et al.* LPS-induced NFκB enhanceosome requires TonEBP/NFAT5 without DNA binding. *Sci. Rep.* **6**, 24921; doi: 10.1038/srep24921 (2016).

## Supplementary Material

Supplementary Information
