# Supplementary material for: LPS-induced NFκB enhanceosome requires TonEBP/NFAT5 without DNA binding
Source: Sci Rep. 2016 Apr 27;6:24921. doi: 10.1038/srep24921 (PMC4847014; doi:10.1038/srep24921)
Supplement: Supplementary Information [file srep24921-s1.doc]

**Supplementary Information**

**LPS-induced NFκB enhanceosome requires TonEBP/NFAT5 without DNA binding**

Hwan Hee Lee^1^, Satoru Sanada^2^, Seung Min An^1^, Byeong Jin Ye^1^, Jun Ho Lee^1^, Young-Kyo Seo^1^, Changwook Lee^1^, Whaseon Lee-Kwon^1^, Christoph Küper^3^, Wolfgang Neuhofer^4^, Soo Youn Choi^1^,*, Hyug Moo Kwon^1^,*

^1^School of Life Sciences, Ulsan National Institute of Science and Technology, Korea; ^2^Division of Nephrology, Japan Community Care Organization Sendai Hospital, Japan; ^3^Institute of Physiology, University of Munich, Germany; ^4^Department of Medicine, Heidelberg University, Germany

**Supplementary Figures**


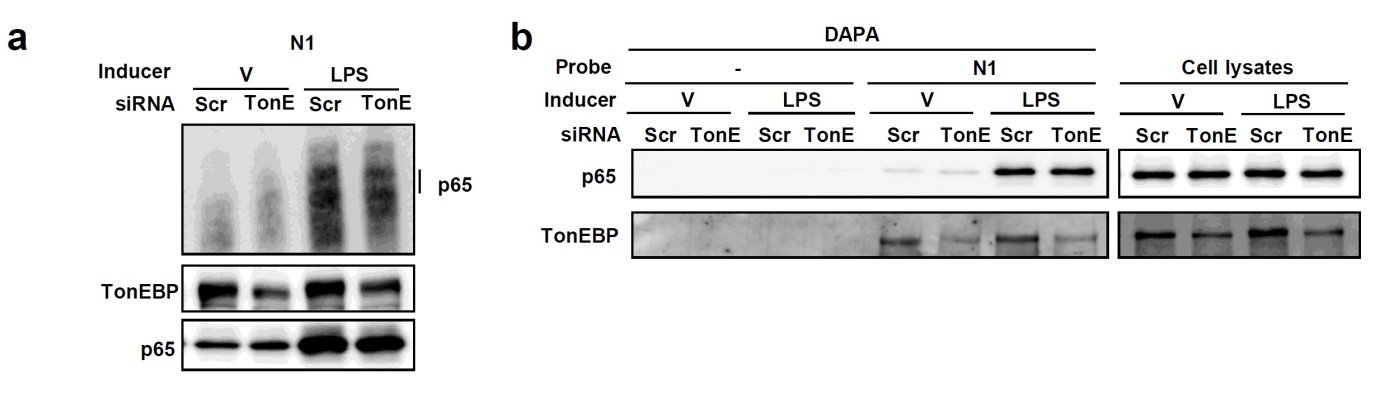


**Supplementary Figure 1 (related to Figure 1). TonEBP knockdown does not affect DNA binding of p65.**

(a) RAW264.7 cells were transfected with TonEBP-targeting siRNA (TonE) or scrambled siRNA (Scr) followed by treatment with LPS or vehicle (V). Nuclear extracts were prepared and EMSA was performed with the N1 probe as in Fig 1b (top). The nuclear extracts were immunblotted for TonEBP and p65 (bottom). (b) DAPA was performed with the N1 probe as in Fig 1e.

**
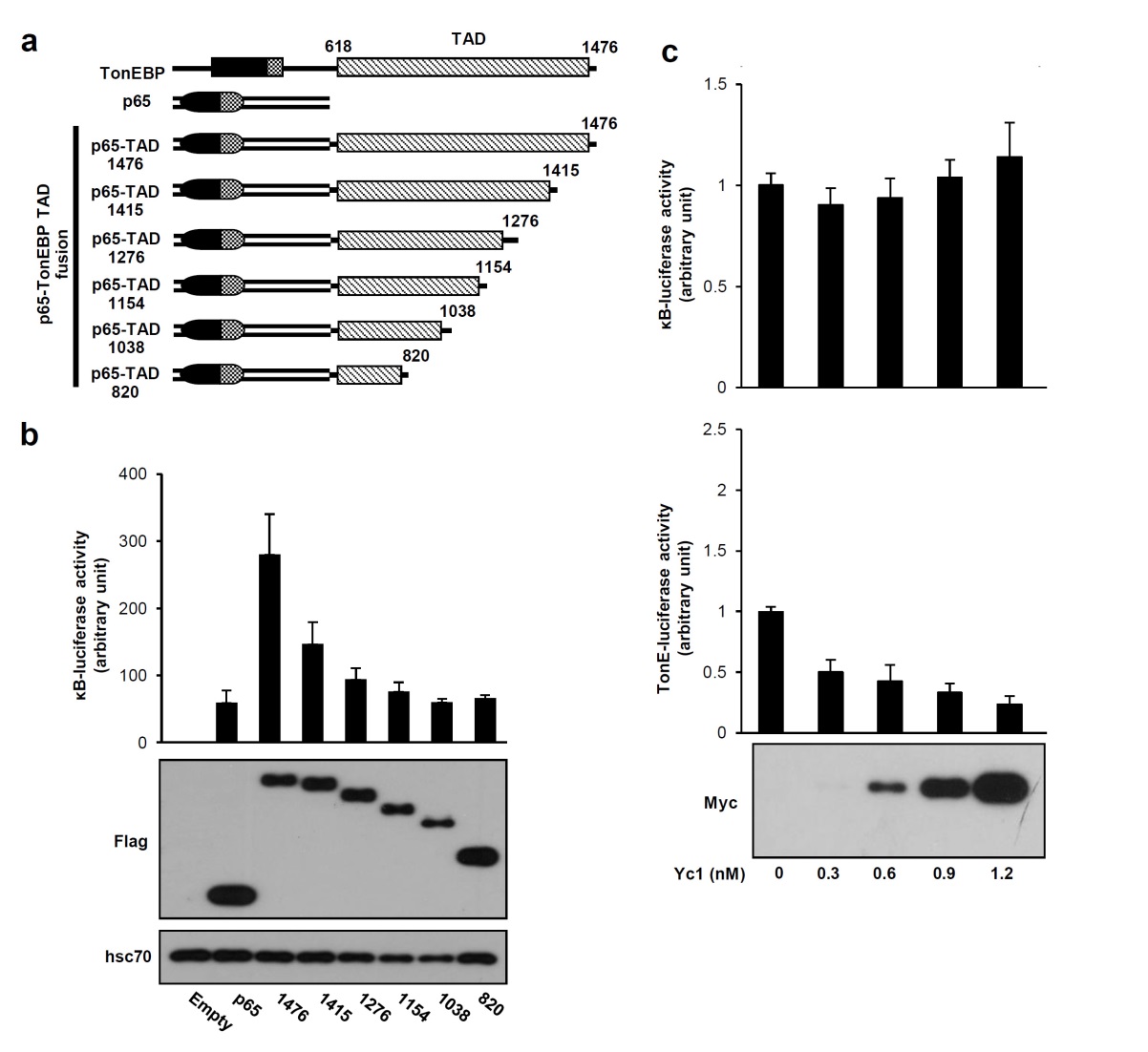
**

**Supplementary Figure 2 (related to Figure 2). The transactivation domain of TonEBP mediates the NFκB activation.**

(a) Schematic representation of the p65-TonEBP fusion constructs. Transactivation domain (TAD) of TonEBP (amino acids 618-1476) and its serial deletions were fused to p65 as shown. (b) COS-7 cells were transfected with Flag-tagged p65 or various p65-TonEBP fusion constructs along with a κB-luciferase reporter construct driven. Cells were stimulated for 6 hours with LPS before measuring luciferase activity. Mean + SD, n = 5. Expression of the p65-TonEBP fusion proteins was examined by immunoblotting for Flag. (c) COS7 cells were transfected with Myc-Yc1 along with a luciferase reporter construct driven by κB or TonE. Cells were stimulated for 6 hours with LPS before measuring κB luciferase activity or stimulated for 6 hours with hypertonicity before measuring TonE luciferase activity. Mean + SD, n = 6.


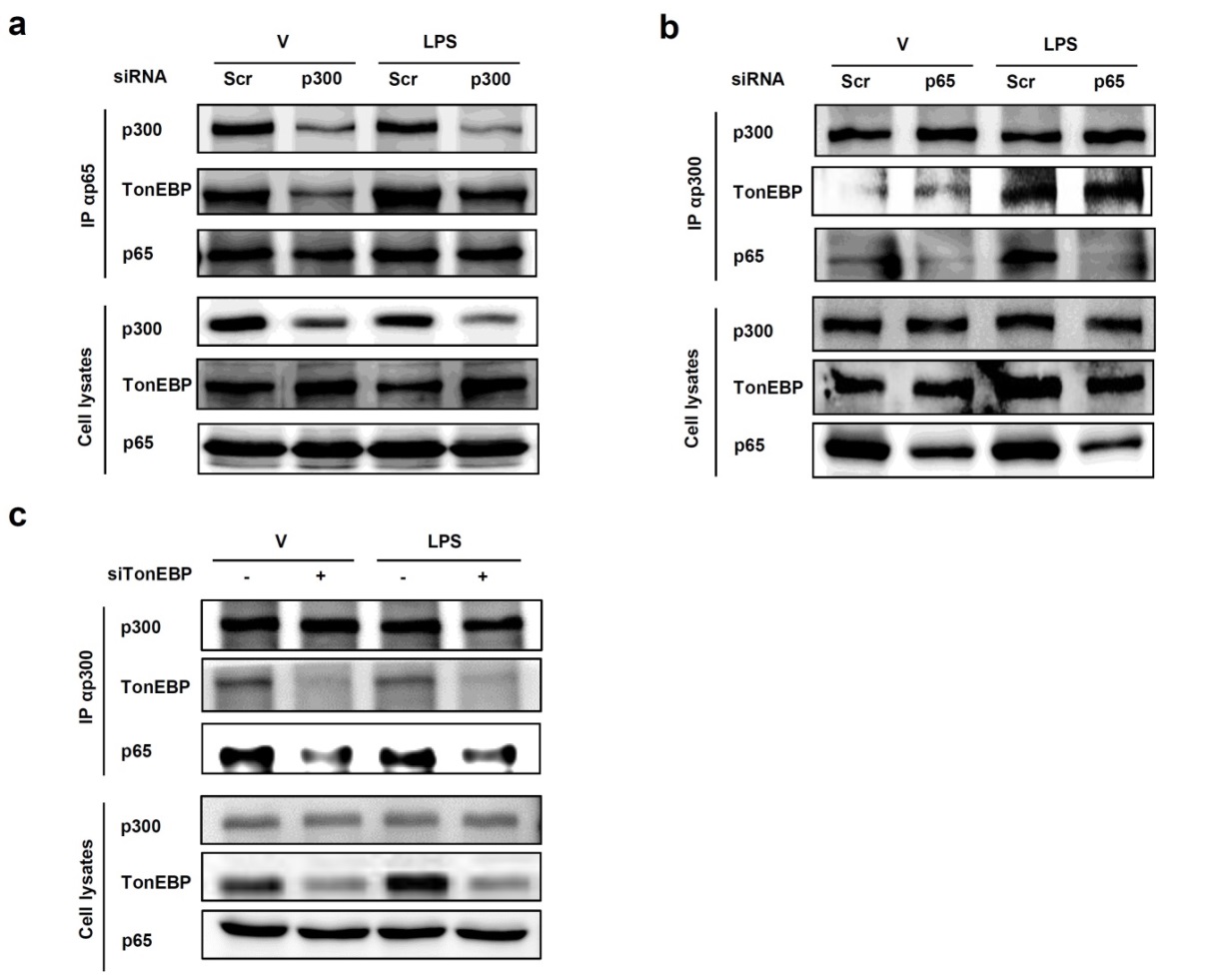


**Supplementary Figure 3 (related to Figure 6). TonEBP-p300 interaction is independent of p65.**

(a) MEF cells were transfected with p300-targeted siRNA or scrambled (Scr) siRNA followed by stimulation with LPS or vehicle (V). Cell lysates were immunoprecipitated (IP) using anti-p65 antibody and immunoblotted for p300, TonEBP and p65. (b) MEF cells transfected with p65-targeted siRNA or scrambled (Scr) siRNA were treated as above. Cell lysates were immunoprecipitated using anti-p300 antibody. (c) MEF cells transfected with TonEBP-targeted siRNA or scrambled (Scr) siRNA were treated as above. Cell lysates were immunoprecipitated using anti-p300 antibody.


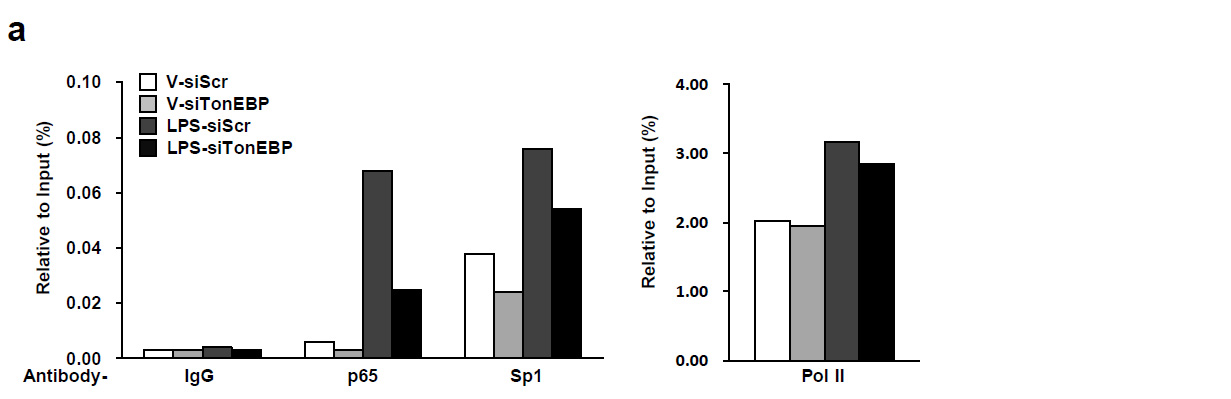


**Supplementary Figure 4 (related to Figure 6). TonEBP deficiency breaks down the LPS-induced NFκB enhancesome assembly on TNFα promoter.**

RAW264.7 cells were transfected with TonEBP-targeted siRNA or scrambled (Scr) siRNA followed by stimulation with LPS or vehicle (V) for 1h. ChIP was performed using normal rabbit IgG, anti-p65 IgG, anti-Sp1 IgG, and anti-Pol ll IgG. The precipitates were quantified for the N1 region using Q-PCR. A representative set of three independent experiments is shown.


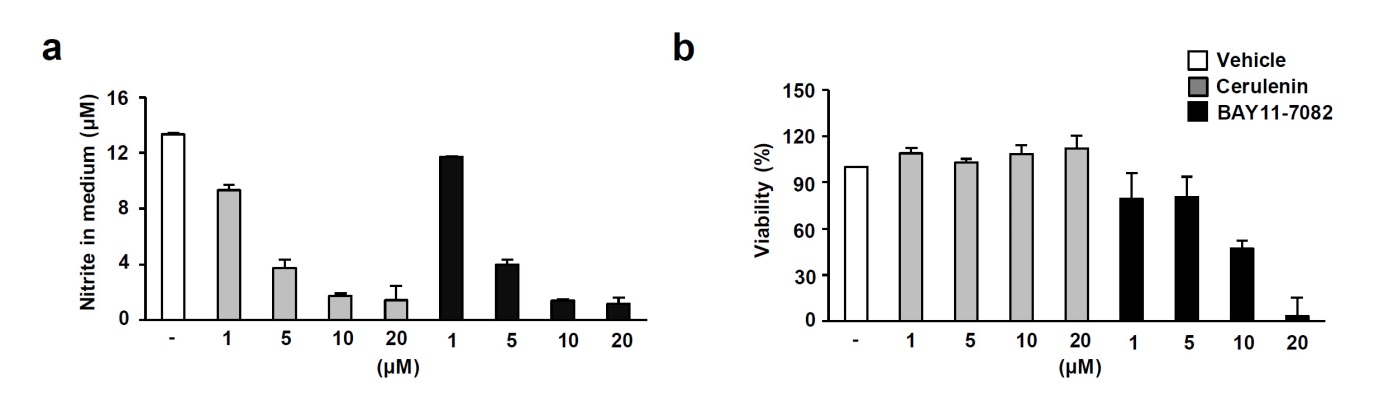


**Supplementary Figure 5 (related to Figure 8). Cerulenin reduces inflammation without cytotoxicity.**

(a) RAW264.7 cells were treated for 1 h with vehicle, or 1 to 20 μM of cerulenin or BAY11-7082 as indicated. The cells were then treated for 24 h with LPS. Nitrite in the media (a) and cell viability (b) were measured. Mean + SD, n = 5.


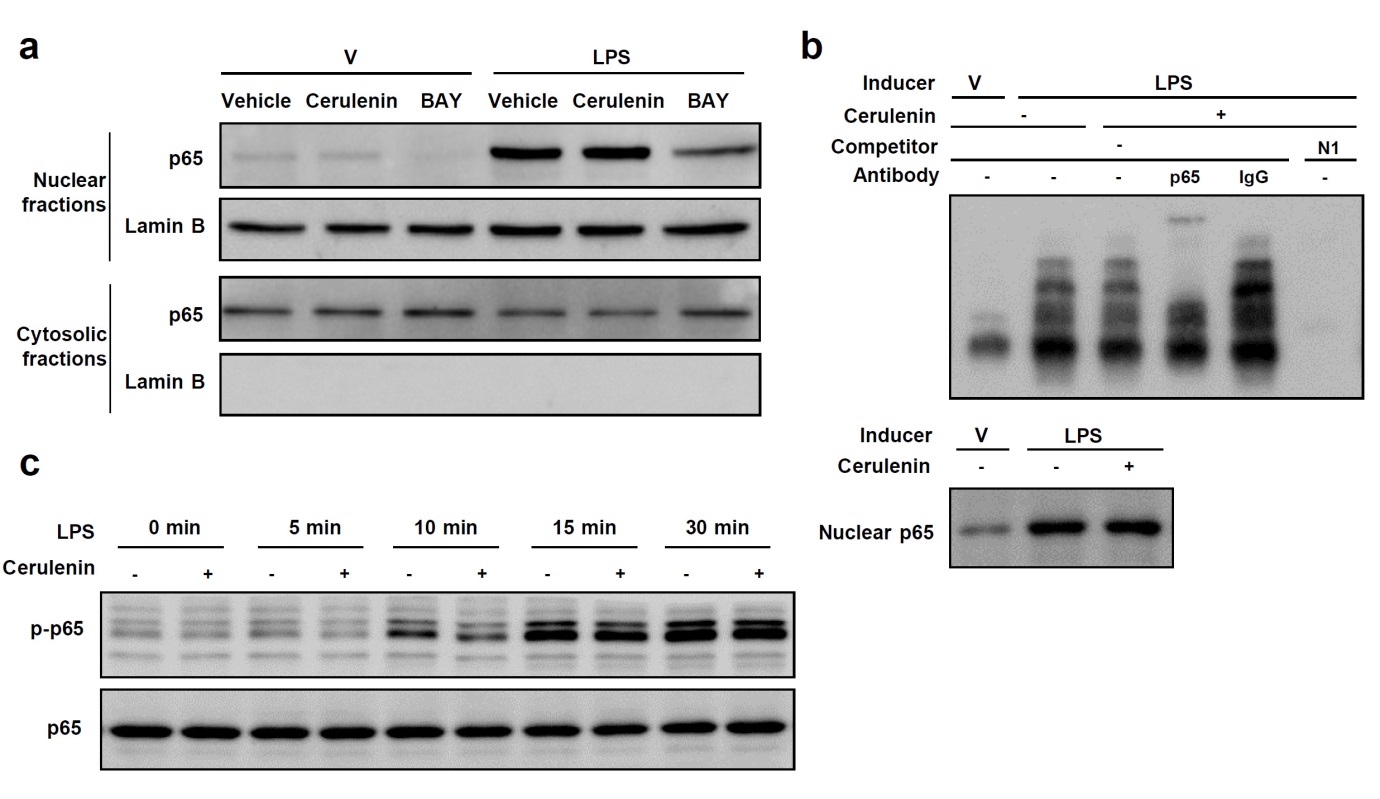


**Supplementary Figure 6 (related to Figure 8). Cerulenin does not affect nuclear localization, DNA binding, and phosphorylation of p65.**

(a) RAW264.7 cells were treated for 1 h with vehicle, cerulenin, or BAY 11-7082 (BAY) followed by 1 h treatment with vehicle (V) or LPS. Nuclear and cytoplasmic fractions were separated and immunoblotted for p65, and lamin B. (b) Cells were pretreated with cerulenin and then LPS as above. EMSA was performed using nuclear extracts and biotin-labeled N1 probe. Where indicated, anti-p65 IgG (p65) or control IgG (IgG) was added to supershift p65-DNA complex. In the last lane, 50 times concentration of unlabeled (cold) N1 was added for competition. Bottom panel shows p65 immunoblot of the nuclear extracts. (c) Vehicle (-) or cerulenin treated cells were incubated with LPS for 0 to 30 min as indicated, and immunoblotted for serine 276 phosphorylated p65 (p-p65) and p65.


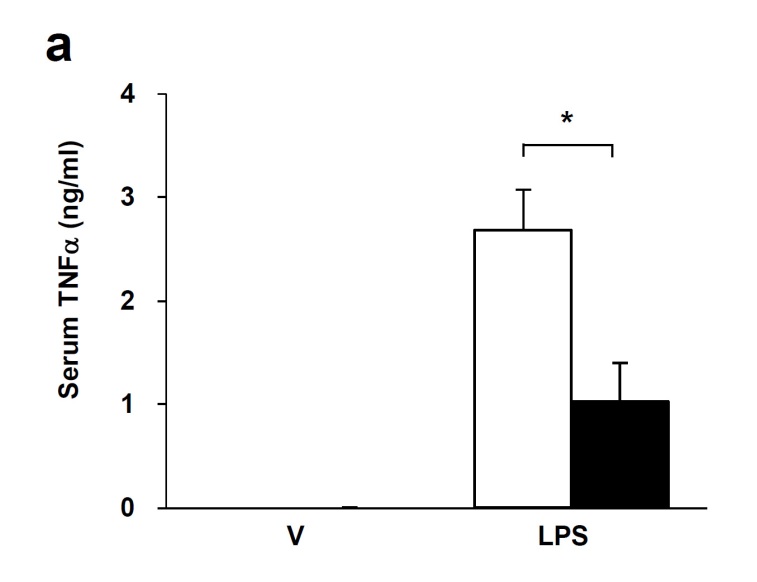


**Supplementary Figure 7 (related to Figure 7). TonEBP haplo-deficient mice showed reduced serum TNFα concentration in response to LPS injection.**

*TonEBP^+/Δ^* mice (solid bars) and their *TonEBP^+/+^* littermates (open bars) were intraperitoneally injected with LPS (60 mg/kg) (LPS) or vehicle (V). After 1 h, TNFα was measured from serum samples. Mean + SD, n = 5. *p<0.05.
